# Single-arm trial to evaluate the efficacy and safety of baclofen in treatment of intractable hiccup caused by malignant tumor chemotherapy

**DOI:** 10.1515/med-2023-0664

**Published:** 2023-03-08

**Authors:** Mengxue Mei, Ming Fang, Ye Mao, He Chen, Long Huang

**Affiliations:** Department of Oncology, The Second Affiliated Hospital of Nanchang University, Nanchang 330006, Jiangxi, China; JiangXi Key Laboratory of Clinical and Translational Cancer Research, Nanchang 330006, Jiangxi, China; Department of Oncology, Yangxin People’s Hospital of Hubei Province, Hubei, China; Jiangxi Key Laboratory of Molecular Medicine, The Second Affiliated Hospital of Nanchang University, Nanchang 330006, Jiangxi, China

**Keywords:** intractable hiccup, malignant tumor, chemotherapy, baclofen, therapeutic effect

## Abstract

Previous studies suggest that baclofen may be useful in the treatment of intractable hiccup caused by chemotherapy. This study was aimed to assess the possible efficacy and safety of baclofen. In total, 65 patients with intractable hiccup caused by chemotherapy were screened. 45 patients with intractable hiccup caused by chemotherapy were finally recruited. Participants in the trial received 10 mg baclofen three times daily for 3 days. The primary outcome measure was cessation of hiccups. Secondary outcome measures included efficacy and adverse events. All 45 patients completed the study. Among them, 41 cases were cured (91.11%, 41/45), 4 cases were relieved (8.89%, 4/45), and the overall effective rate was 100% (45/45). Furthermore, the median remission time was 2(1, 9) times, the median cure time was 2(1, 9) times, the remission rate of one-time was 13.33% (6/45), the remission rate of two-time was 53.33% (24/45), and 2 cases (4.44%, 2/45) relapsed after drug withdrawal. No serious adverse events were documented. Only 1 case (2.22%) had grade 2 fatigue and 2 cases (4.44%) had grade 1 sleepiness. Baclofen is safe and effective in the treatment of intractable hiccup caused by chemotherapy of malignant tumor.

## 1 Introduction

Chemotherapy is an important means to treat malignant tumors. Common chemotherapy drugs include cisplatin, paclitaxel, etc. Cisplatin can combine with the DNA of tumor cells, causing cross-linking, thus destroying the DNA function of tumor cells and inhibiting and killing the proliferation of tumor cells. Paclitaxel can break the balance of tubulin and tubulin dimer, induce and promote tubulin polymerization, thus stabilizing microtubules, inhibiting tumor cell division and triggering cell apoptosis [1]. However, chemotherapy drugs can not only kill cancer cells, but also damage normal cells, leading to chemotherapy side effects in patients [2].

Hiccup is a common side effect of chemotherapy in clinic, mainly due to stimulation of central nervous system, which leads to paroxysmal spasm of unilateral or bilateral diaphragm. According to previous findings, about 2% of patients with malignant tumors have intractable hiccups during chemotherapy, and most patients will have symptoms such as dyspnea, vomiting, and nausea, which will affect the chemotherapy effect [3]. At present, clinically, intractable hiccups are usually treated with gastric motility drugs and sedative drugs, such as metoclopramide and chlorpromazine, among which metoclopramide is a dopamine D2 receptor antagonist, which can act on dopamine receptors in the emetic chemoreceptor area of the medulla oblongata and raise the threshold. At the same time, it can also directly inhibit the chemosensory area of medulla oblongata, and reduce the excitatory impulse to diaphragm [4,5]. Chlorpromazine belongs to the first generation of phenothiazine antipsychotics, which can block the dopamine receptor in the cerebral cortex pathway of midbrain limbic diseases and inhibit the dopamine receptor in the chemically sensitive area of medulla oblongata, so as to inhibit vomiting center, produce anti-emesis effect, and improve the clinical symptoms of patients [6]. However, due to the great differences in clinical individuals, different curative effects, long drug onset time, and high risk of adverse reactions, the clinical treatment effect is average. Therefore, it is still necessary to actively explore the treatment scheme that can effectively improve the intractable hiccup caused by chemotherapy for malignant tumors.

Caffeine belongs to nervous system medicine, which is a muscle relaxant acting on the spinal cord and has a positive effect on relieving reflex muscle spasm [7]. Baclofen is effective in treating refractory gastroesophageal reflux cough, which can effectively relieve clinical symptoms [8]. At present, FDA and CFDA have not approved baclofen’s indication for treating intractable hiccup caused by chemotherapy of malignant tumor. In view of this, this study will analyze the feasibility of baclofen in treating intractable hiccup caused by chemotherapy of malignant tumor from the aspects of therapeutic effect and safety.

## 2 Methods

### 2.1 Study population

In this study, 65 patients with intractable hiccup caused by chemotherapy of malignant tumor were screened from July 2020 to July 2022. Because of severe complications shown in the exclusion criteria and potential confounding factors of intractable hiccup, 20 candidates were excluded from the study, and the remaining 45 patients were included in this study. There were 44 males and 1 female. The median age is 64 (37,75) years; PS score 0–2 points. Types of malignant tumors: 11 cases of liver cancer, 10 cases of lung cancer, 7 cases of colorectal cancer, 4 cases of gastric cancer, 3 cases of esophageal cancer, 2 cases of nasopharyngeal cancer, 1 case of spinal cord tumor, 1 case of glioblastoma, 1 case of thymic adenocarcinoma, 1 case of laryngeal phosphorus cancer, 1 case of pancreatic cancer, 1 case of isthmus carcinoma, 1 case of bladder carcinoma, and 1 case of maxillary sinus carcinoma. Hiccup nature: 30 cases were persistent and 15 cases were intermittent. Severity of hiccup: severe in 19 cases, moderate in 17 cases, and mild in 9 cases. Complications: vomiting in 27 cases and sleep disorder in 22 cases. The median period of intractable hiccup is 1 (1,15). There were 36 cases of first-line chemotherapy, 3 cases of second-line chemotherapy, 4 cases of third-line chemotherapy, and 2 others. Intravenous chemotherapy was used in 35 cases, interventional chemotherapy in 6 cases, and oral chemotherapy in 4 cases. The median time from chemotherapy to hiccup was 21.00 h (12.00 h, 24.00 h).

Inclusion criteria: (1) Malignant tumors were all confirmed by pathological examination; (2) Intractable hiccup meets the relevant diagnostic criteria, namely, hiccups lasting longer than 1 month [9]; (3) All patients received chemotherapy; (4) Hiccup occurred newly within 24 h after chemotherapy; (5) Physical condition (PS) score [10]: 0–2 points; (6) Signed informed consent form. Exclusion criteria: (1) Patients complicated with severe cardiac and pulmonary insufficiency; (2) Those complicated with gastrointestinal bleeding; (3) Those with coagulation dysfunction; (4) Those complicated with severe liver and renal dysfunction; (5) Intractable hiccup caused by other factors, such as cerebral infarction and brain trauma; (6) Those with combined blood diseases. Written informed consent was obtained from all patients. This study was approved by the Ethics Committee of the Second Affiliated Hospital of Nanchang University, and conducted according to the Declaration of Helsinki.

### 2.2 Treatment

The patients were treated with baclofen tablets (Ningbo Tianheng Pharmaceutical Co., Ltd, Fu’an Pharmaceutical Group, specifications: 10 mg*10 s, GuoYaoZhunZi H19980103) when hiccup occurred newly within 24 h after chemotherapy, at 10 mg/time, 3 times/day orally for 3 days. The daily dosage and frequency of baclofen were determined according to the published results and real-world clinical use [11]. During the treatment, no other mediations or treatments were received among patients.

### 2.3 Study outcomes

(1) Clinical efficacy: Cure: the hiccup completely stopped 3 days after treatment. Remission: the attack times of hiccup were obviously reduced, which is more than 50% lower than that before treatment. Invalid: the above criteria are not met or the disease aggravates [12]. Total effective rate = (cured + relieved) cases/total cases × 100%. The patient’s median response time, the median cure time, and the one-time medication response rate were recorded. (2) Adverse reactions and recurrence: adverse reactions including fatigue (conscious fatigue and limb weakness), drowsiness (excessive daytime sleep or sleep onset), tremors (rhythmic and alternating swinging movements), and hiccup recurrence after drug discontinuation were recorded during the treatment.

### 2.4 Statistical analysis

SPSS 25.0 software was used, and GraphPad Prism 8.0 software was used to draw the patient’s medication times and remission after medication.

## 3 Results

### 3.1 Clinical efficacy

**Figure 1:**
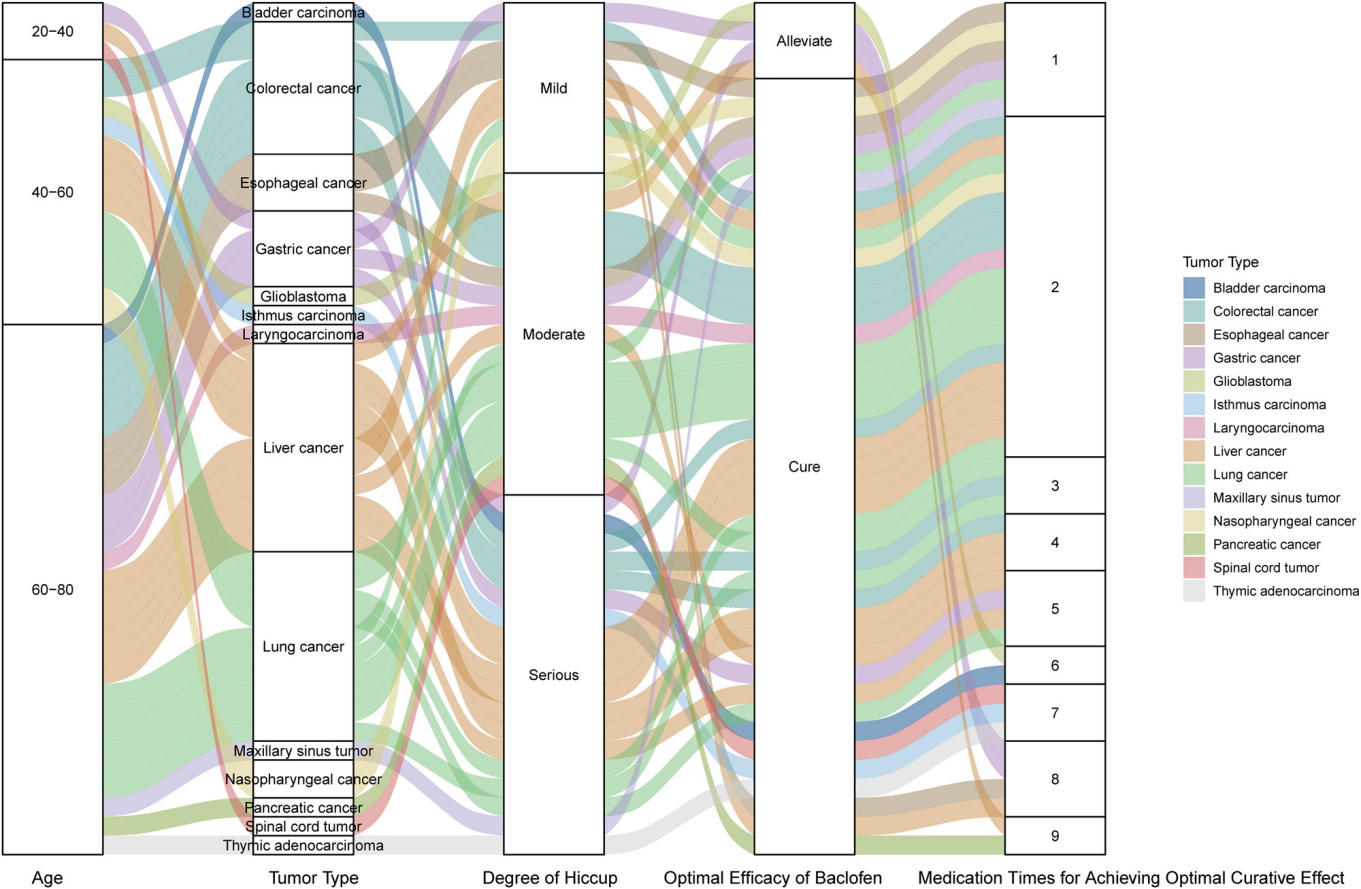
Sankey plot showing the relationships among patients’ characteristics.

All the 45 patients completed the treatment according to the plan. There were 41 cured cases (91.11%), 4 relieved cases (8.89%), and the total effective rate was 100% (45/45). The improvement rates of one-time medication and two-time medication were 13.33% (6/45) and 53.33% (24/45), respectively. The median response time was 2 (1,9) times, and the median cure time of 41 cured patients was 2 (1,9) times (Table 1, Figure 1).

**Table 1:** Baseline data and treatment of patients

Patient number	Age	Disease	Chemotherapy regimens	Degree of hiccup	Optimal efficacy of baclofen	Medication times for achieving optimal curative effect	Median times for response to baclofen
1	67	Lung cancer	Etoposide + carboplatin	Moderate	Cure	2	1
2	37	Gastric cancer	Paclitaxel + S-1	Serious	Cure	5	2
3	34	Spinal cord tumor	Temozolomide	Moderate	Cure	7	3
4	65	Gastric cancer	Oxaliplatin + paclitaxel + S-1	Mild	Alleviate	8	2
5	67	Gastric cancer	Docetaxel + 5-fu + oxaliplatin	Serious	Alleviate	8	2
6	46	Glioblastoma	Temozolomide	Moderate	Alleviate	6	2
7	69	Esophageal cancer	S-1	Mild	Cure	8	2
8	69	Liver cancer	Ifosfamide + doxorubicin	Serious	Cure	2	1
9	57	Liver cancer	Oxaliplatin + 5-fu	Serious	Cure	4	2
10	56	Lung cancer	Pemetrexed + cisplatin	Serious	Cure	5	1
11	64	Lung cancer	Pemetrexed	Mild	Cure	2	1
12	57	Lung cancer	Paclitaxel	Moderate	Cure	2	2
13	65	Lung cancer	Etoposide + carboplatin	Moderate	Cure	1	1
14	62	Esophageal cancer	Paclitaxel + carboplatin	Mild	Cure	1	1
15	69	Lung cancer	Paclitaxel + nedaplatin	Moderate	Cure	3	2
16	64	Thymic adenocarcinoma	S-1	Serious	Cure	7	2
17	74	Laryngocarcinoma	Docetaxel + nedaplatin	Moderate	Cure	2	1
18	22	Liver cancer	Oxaliplatin + 5-fu + pirarubicin	Moderate	Alleviate	9	3
19	53	Liver cancer	Oxaliplatin + 5-fu + pirarubicin	Serious	Cure	2	1
20	42	Nasopharyngeal cancer	Paclitaxel + nedaplatin	Mild	Cure	1	1
21	50	Nasopharyngeal cancer	Paclitaxel + nedaplatin	Mild	Cure	2	1
22	64	Liver cancer	Gemcitabine + cisplatin	Serious	Cure	5	2
23	58	Lung cancer	Docetaxel	Moderate	Cure	2	1
24	72	Colorectal cancer	Oxaliplatin + 5-fu + bevacizumab	Moderate	Cure	2	2
25	65	Pancreatic cancer	Paclitaxel + Gemcitabine	Moderate	Cure	9	1
26	54	Colorectal cancer	Oxaliplatin + irinotecan + 5-fu	Mild	Cure	2	2
27	58	Colorectal cancer	Oxaliplatin + 5-fu + bevacizumab	Serious	Cure	2	1
28	53	Isthmus carcinoma	Docetaxel + nedaplatin	Serious	Cure	7	4
29	68	Bladder carcinoma	Paclitaxel + Tirelizumab	Serious	Cure	6	3
30	71	Colorectal cancer	Oxaliplatin + 5-fu + bevacizumab	Serious	Cure	4	2
31	42	Liver cancer	Oxaliplatin + 5-fu + pirarubicin	Serious	Cure	4	3
32	73	Liver cancer	Oxaliplatin + 5-fu + pirarubicin	Moderate	Cure	5	2
33	75	Colorectal cancer	Oxaliplatin + capecitabine	Moderate	Cure	2	1
34	68	Esophageal cancer	Paclitaxel	Moderate	Cure	1	1
35	72	Liver cancer	Oxaliplatin + 5-fu + pirarubicin	Mild	Cure	8	2
36	64	Lung cancer	Etoposide + Carboplatin	Serious	Cure	3	1
37	63	Maxillary sinus tumor	Doxorubicin + cyclophosphamide	Serious	Cure	1	1
38	65	Colorectal cancer	Oxaliplatin + 5-fu	Serious	Cure	3	2
39	65	Liver cancer	Oxaliplatin + 5-fu + pirarubicin	Serious	Cure	2	1
40	68	Liver cancer	Oxaliplatin + 5-fu + pirarubicin	Mild	Cure	2	1
41	58	Lung cancer	Paclitaxel + carboplatin	Serious	Cure	2	2
42	75	Lung cancer	Paclitaxel + carboplatin	Moderate	Cure	2	1
43	72	Colorectal cancer	Irinotecan + 5-fu + Bevacizumab	Moderate	Cure	2	1
44	69	Gastric cancer	Oxaliplatin + capecitabine	Moderate	Cure	1	2
45	58	Liver cancer	Oxaliplatin + 5-fu	Serious	Cure	2	1

### 3.2 Adverse reaction and recurrence rate during chemotherapy

One case (2.22%) experienced fatigue (the patient experienced fatigue after three treatments, manifested as conscious fatigue and limb weakness, but the symptoms were mild and they spontaneously disappeared without intervention). Two cases (4.44%) experienced Grade 1 somnolence (the patient experienced somnolence after two treatments and manifested as excessive daytime sleep or sleep episodes). Two cases (4.44%) relapsed (1 cured patient relapsed into hiccup 3 days after drug discontinuation, and the another cured patient relapsed into hiccup 5 days after drug discontinuation) (Table 2).

**Table 2:** Characteristics of patients who had recurrence or adverse reactions

Patient number	Age	Sex	Disease	Grade	PS score	Chemotherapy regimen	Degree of hiccup	Relapse after baclofen withdrawal	Fatigue	Somnolence
24	72	Male	Colorectal cancer	cT3N + M1b ⅣB	2	Oxaliplatin + 5-fu + bevacizumab	Moderate	No	No	Yes
33	75	Male	Colorectal cancer	T3aN0M0	1	Oxaliplatin + capecitabine	Moderate	No	No	Yes
34	68	Male	Esophageal cancer	cT3N1M0 ⅢA	1	Paclitaxel	Moderate	Yes	Yes	No
42	75	Male	Lung cancer	cT4N2M0 ⅢB	1	Paclitaxel + carboplatin	Moderate	Yes	No	No

## 4 Discussion

Intractable hiccup, a common toxic and side effect of chemotherapy, is caused by multiple involuntary spastic contractions of the transverse diaphragm and intercostal muscles. Hiccup usually begins with sudden inspiration and ends with glottic closure. This rapid movement, accompanied by uncontrollable inhalation, causes the epiglottis to suddenly close the respiratory tract, thus making a short, loud sound [12,13]. Intractable hiccup refers to the case that the spasm persists for more than 48 h, which can lead to laceration of the test tube at the cardia part and the gastric fundus mucosa as the disease progresses, reducing the safety of chemotherapy [14,15].

Baclofen, a gamma-aminobutyric acid (GABA)-derived organism, inhibits the release of excitatory amino acids by exciting GABA-β receptor, thereby reducing the reflex potentials at single or multiple synapses in the spinal cord and between the posterior roots of the spinal cord, thereby playing a role in relaxing the skeletal muscle and relieving spasm [16,17]. Colorado and Decker [18] found that baclofen can help alleviate the symptoms of persistent hiccup in stroke patients with less adverse reactions. However, there is only a small sample size retrospective study on clinical hiccup caused by therapeutic tumor chemotherapy with baclofen. The clinical efficacy and safety of baclofen in the treatment of hiccup caused by malignant tumor chemotherapy are still unclear. At present, all the guidelines fail to take baclofen as a commonly used drug for the treatment of hiccup.

The results of this study showed that the total effective rate of baclofen in the treatment of hiccup caused by chemotherapy for malignant tumors was 100%, indicating that baclofen could effectively improve the treatment effect of patients with intractable hiccup caused by chemotherapy for malignant tumors. The reason for this analysis was that according to the research results of Zhang et al. [19], the total effective rate of 28 patients with intractable hiccup after primary liver cancer intervention was 92.86% (26/28) after treatment with baclofen. Similar to the research results, baclofen may play an important role in the treatment of intractable hiccup. Chemotherapy for malignant tumors can directly stimulate the vagus nerve, resulting in increased vagal function tension and diaphragmatic spasm. Ehret et al. [20] found that the incidence rate of intractable hiccup in patients with advanced cancer is about 3.9–4.98%, which belongs to the common toxic and side effects of chemotherapy in clinic. Intractable hiccup can seriously affect patients’ eating and rest, causing patients’ nutritional level to decrease, electrolyte disorders, depression, insomnia, and weakened immune function, thus affecting the patient’s treatment effect. At the same time, according to relevant research results, severe toxic and side effects can lead to patients’ resistance to chemotherapy, or even abandoning chemotherapy, thus affecting the efficacy of chemotherapy treatment of patients and shortening the survival time [21]. Baclofen, on the other hand, can act on GABA-β receptor of spinal motor neurons, inhibit synaptic reflex, and reduce excitatory synaptic sites as well as the reflex potential between hormone dorsal root and dorsal root, thereby playing a role in relaxing the skeletal muscle, improving clinical symptoms and chemotherapy comfort of patients [22].

The results of this study have shown that patients had a high rate of symptom relief after one dose of medication, indicating that baclofen has the characteristics of quick effect and strong drug effect. The reason for this analysis is that baclofen can be rapidly and completely absorbed through the gastrointestinal tract. According to the results of relevant studies, a single oral dose of 10, 20, and 30 mg baclofen can reach the peak plasma drug concentration in 0.5–1.5 h, thus exerting the drug effect rapidly [23].As one of the main inhibitory neurotransmitters in the brain and spinal cord of the central nervous system, GABA content is high, and GABA is a neurotransmitter for about 20–40% of synapses in the brain. By exciting the GABA-β receptor, baclofen inhibits the release of excitatory amino acids, and promotes the outflow of potassium and calcium ions in neurons to produce super-effect, reducing the transmission of synaptic reflex and reducing the activity of α-motor neurons, thereby relieving skeletal muscle spasm, reducing diaphragmatic muscle tension, improving the clinical symptoms of patients, improving the clinical efficacy, and reducing the frequency of drug administration [24]. Baclofen, a similar neurotransmitter with inhibitory effect on nerve conduction, is able to inhibit the single-synaptic and multi-synaptic reflex of the central nervous system, highly polarize the afferent end, and inhibit the central nervous system, thereby relieving the spasm caused by the damage of upper motor neurons, myoclonus, and muscle tremor, and reducing the frequency of hiccup attack [25].

In this study, there was only one case of fatigue and two cases of drowsiness. Besides, the patients’ adverse reaction symptoms were mild and they spontaneously disappeared without intervention. In previous literature, the adverse effects of oral baclofen could impact more than 25% of patients, and the symptoms usually included somnolence, nausea, and dizziness [11]. The rate of adverse reaction was lower in our study compared to published results, and the discrepancy could be explained as follows: (1) the subjects in our study were patients suffering from chemotherapy, and the homogeneous population could show less adverse events. (2) The dosage of baclofen was lower compared to other studies, in which the dosage usually exceeded 50 mg/day. It can be seen that the safety of baclofen treatment was high. The reason for this analysis was that the plasma elimination half-life of baclofen was 3–4 h, which was mostly excreted in prototype. Within 72 h, 75% of baclofen was excreted by kidney, with less drug retained in the body. At the same time, the main metabolite of baclofen is (p-chlorophenyl)-γ-hydroxybutyric acid without pharmacological activity, so the risk of adverse reactions is low. However, the safety of the specific drug needs further clinical research [26]. GABA is converted into baclofen by attaching p-chlorobenzene cluster to β carbon atom, so that through the blood–brain barrier, the skeletal muscle spasm caused by damaged pyramidal tract is relieved, the muscle tension is reduced, and the clinical symptoms of the patient are improved [27].

Our study had several strengths. We first conducted an explorative study on the efficacy and safety of baclofen in treatment of intractable hiccup caused by malignant tumor chemotherapy, and offered original data for the following randomized controlled trials. In addition, to the best of our knowledge, this is the largest sample size available for a prospective study of baclofen in the treatment of intractable hiccup induced by chemotherapy for malignant tumors. However, limitation also existed in our study. First, our study was a single-center study, and enrolled patients may be biased. Second, our study had a small number of enrolled cases, and the distribution of patients’ gender was imbalanced. In addition, there was a lack of randomized control group. Since we aimed to provide an explorative result of baclofen treatment, the results of this study need to be verified by prospective randomized controlled trials with larger sample size.

In summary, baclofen has obvious effects and less side effects in the treatment of intractable hiccup caused by chemotherapy for malignant tumors.
